# Clinical Features and Risk Factors of Active Tuberculosis in Patients with Behçet's Disease

**DOI:** 10.1155/2020/2528676

**Published:** 2020-11-24

**Authors:** Yaxu Liu, Lifan Zhang, Ziyue Zhou, Luxi Sun, Baotong Zhou, Xiaoqing Liu, Wenjie Zheng

**Affiliations:** ^1^Department of Infectious Diseases, Peking Union Medical College Hospital, Chinese Academy of Medical Sciences & Peking Union Medical College, Beijing 100730, China; ^2^Peking Union Medical College Hospital, Chinese Academy of Medical Science, Clinical Epidemiology Unit, International Epidemiology Network, Beijing 100730, China; ^3^Chinese Academy of Medical Sciences and Peking Union Medical College, Centre for Tuberculosis Research, Beijing 100730, China; ^4^Department of Rheumatology, Peking Union Medical College Hospital, Chinese Academy of Medical Sciences & Peking Union Medical College, The Ministry of Education Key Laboratory, National Clinical Research Center for Dermatologic and Immunologic Diseases, Beijing 100730, China

## Abstract

To investigate the clinical features and potential risk factors of active tuberculosis (ATB) in Behçet's disease (BD), we conducted a case-control study on hospitalized BD patients in our institute from 2010 to 2019. BD patients with ATB were enrolled as the case group. The control group was selected by random number sampling from the remaining BD patients, including those with latent tuberculosis infection, previous tuberculosis, or without tuberculosis. Finally, we reviewed 386 BD patients and identified 21 (5.4%) ATB cases, including four (19.0%) microbiologically confirmed and 17 (81.0%) clinically diagnosed. We found that BD patients with ATB were more prone to have systemic symptoms (fever, night sweating, and unexplained weight loss) and/or symptoms related to the infection site. Multivariate logistic regression analysis revealed that erythrocyte sedimentation rate (ESR) > 60 mm/h (OR = 13.710, 95% CI (1.101, 170.702)), increased IgG (OR = 1.226, 95% CI (1.001, 1.502)), and positive T-SPOT.TB (OR = 7.793, 95% CI (1.312, 48.464), for 24-200 SFC/10^6^PBMC; OR = 17.705 95% CI (2.503, 125.260), for >200 SFC/10^6^PBMC) were potential risk factors for ATB in BD patients. Our study suggested that when BD patients have systemic symptoms with significantly elevated TB-SPOT, the diagnosis of ATB should be considered.

## 1. Introduction

Behçet's disease (BD) is a systemic vasculitis prevalent in the areas along the Ancient Silk Road such as Turkey, Iran, China, and Japan [1]. In China, the prevalence of BD is estimated to be 14 in 100 000 [2]. It has been noticed that BD is closely related to tuberculosis (TB) [3, 4], which is also prevalent in China [5].

TB infection may trigger the pathogenesis of BD [6, 7], and BD patients are at high risk of active tuberculosis (ATB) infection [8, 9]. The treatment of BD generally includes glucocorticoid, immunosuppressant, and biologic agents [10–12]. Once a BD patient is infected with ATB, the immunosuppressive therapy would be reduced or suspended [13], resulting in the BD deterioration and increased risk of developing immune reconstitution inflammatory syndrome [14]. If the ATB is not promptly diagnosed or properly treated, the intensive immunosuppressive therapy may lead to disseminated TB and severe consequences [15]. Thus, understanding the clinical features and risk factors of ATB in patients with BD is of great importance.

To date, related studies are very limited. Lin et al. reported the clinical features of ATB in 10 BD patients, but the BD sample size for the study was only 37, and the statistical analysis was not conducted [16]. Liu compared the clinical features of 36 BD patients with ATB and 407 BD patients without TB. Liu discovered that ATB patients were more prone to fever, arthritis, thrombosis, and elevated IgG [17]. However, patients with latent tuberculosis infection (LTBI) or previous tuberculosis (PTB) were not included in the study. Moreover, to the best of our knowledge, there is no study investigating the risk factor of ATB in BD patients. Therefore, a case-control study was conducted to investigate the clinical features and explore the risk factors of ATB in BD patients.

## 2. Materials and Methods

### 2.1. Participants

Medical records of all hospitalized patients diagnosed with BD in Peking Union Medical College Hospital (PUMCH) from January 1, 2010, to June 30, 2019, were retrospectively reviewed. All patients fulfilled the 2013 International Criteria for Behçet's Disease (ICBD) [18]. Patients suspected of tuberculosis infection underwent careful examinations, including pathological examinations (culture and acid-fast staining of sputum and other available samples like pleural effusion), a biopsy of the suspected organs (colonoscopy of the colorectal lesions, the surgical sample of the vertebra), and immunological examinations (T-SPOT.TB test), for the evidence of tuberculosis infection as well as the exclusion of other common infections in BD like bacteria and CMV. The categorization of TB was based on the 2000 criteria [19] and those applied in previous studies [20, 21] (see Table S1 in the Supplementary Material online). Two experienced experts of infectious diseases were asked to review the cases and check the diagnosis of ATB independently. Only confirmed by both experts would the clinical diagnosis of ATB be accepted. All patients diagnosed with BD and ATB were enrolled as the case group. The remaining patients with BD were ranked according to the date of admission, and the random samples were chosen using random number tables as the control group.

### 2.2. Data Collection

**Figure 1:**
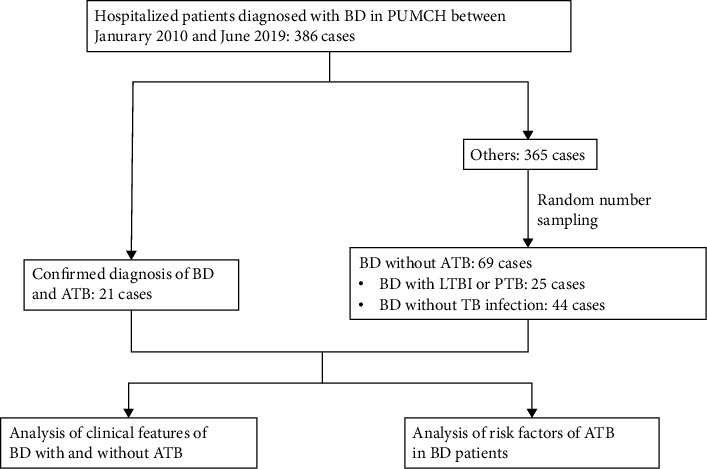
Flowchart of the study. BD: Behçet's disease; PUMCH: Peking Union Medical College Hospital; ATB: active tuberculosis; LTBI: latent tuberculosis infection; PTB: previous tuberculosis.

The demographic features (age and sex), past medical history (the course and treatment of BD before hospitalization, previous infection of TB, etc.), current treatment (glucocorticoid dosage, immunosuppressant, etc.), BD systemic involvement, TB-related systemic symptoms (fever, cough, etc.), laboratory results (complete blood cell count, T-SPOT.TB, etc.), and comorbidities of the patients were collected and analyzed. The flowchart of the study was shown in Figure 1.

### 2.3. Ethic Review

This study complied with the Declaration of Helsinki and was approved by the Ethics Committee of PUMCH (ethics approval number: S-715). Informed consent was obtained from all patients for being included in the study.

### 2.4. Statistical Analyses

Continuous variables were examined by the Kolmogorov-Smirnov test, and those with normal distribution were expressed as mean ± standard deviation (SD), while those without were described as median and interquartile range (IQR). Categorical variables were presented as numbers and percentages. Comparisons of continuous variables were performed using Student's *t*-test when they were in normal distribution, and Mann–Whitney *U*-test when they were not. Categorical data were compared using the Chi-squared test or Fisher's exact test. *p* < 0.05 was considered to be statistically significant.

Variables with *p* < 0.1 in the univariate analysis were considered candidate risk factors, and those with clinical significance were evaluated with stepwise binary logistic regression analysis (inclusion threshold *p* < 0.05, exclusion threshold *p* > 0.1). The odds ratio (OR) of the risk factors and its 95% confidential interval (CI) were calculated. All statistical analyses were performed with SPSS 16.0 (SPSS Inc., USA).

## 3. Results

### 3.1. General Data

Three hundred eighty-six hospitalized patients were diagnosed with BD in PUMCH from January 2010 to June 2019. Twenty-one (5.4%) of them were confirmed with the diagnosis of ATB, including four microbiologically confirmed and 17 clinically diagnosed. The control group consisted of 69 patients, including 25 (36.2%) with LTBI or PTB and 44 (63.8%) without TB.

### 3.2. Clinical Features of BD with ATB

Four of the 21 ATB patients were microbiologically confirmed while 17 were clinically diagnosed (see Table S2 in the Supplementary Material online). Sixteen (76.2%) patients had pulmonary TB, four (19.0%) had extrapulmonary TB, and one patient's infection site could not be identified. Four patients (19.0%) had more than one organ involved. The involved organs other than the lung included the gastrointestinal tract (2, 9.5%), bones (2, 9.5%), lymph nodes (1, 4.8%), larynx (1, 4.8%), peritoneum (1, 4.8%), and pericardium (1, 4.8%).

Patients with ATB were more likely to develop systematic symptoms including fever (18, 85.7%), night sweating (8, 38.1%), and unexplained weight loss (13, 61.9%). Meanwhile, many ATB patients also had symptoms related to the site of infection. For example, pulmonary ATB patients had a productive cough; osteal ATB patients had bone pain and restricted range of motion; lymphatic ATB patients had lymphadenopathy and sinus formation; pharyngeal ATB patients had mucosal erosions, ulcers, and tubercles; and gastrointestinal ATB patients had abdominal pain, diarrhea, and/or constipation.

All ATB patients showed abnormalities in chest radiology, including nodules, cord, or patches, predominantly in the upper and medial lobes. Some other abnormalities can also be revealed by CT scan, such as lymph node enlargement with a low-density core in lymphatic ATB, vertebral damage in osteal ATB, and swelling of gastrointestinal walls in gastrointestinal ATB. The pericardial ATB patients also showed fibrinous pericardial effusion on echocardiogram.

All BD patients with ATB had clinical improvement after anti-TB treatment. Moreover, with intensive treatment of BD following the control of ATB, patients had achieved long-term stability of their disease condition during the follow-up period.

### 3.3. Clinical Comparison of BD Patients with and without ATB

#### 3.3.1. Past Medical History and Medication

More ATB patients had evidence of previous TB infection (13 (61.90%) vs. 17 (24.64%), *p* = 0.002). The duration of previous glucocorticoid use was shorter in the ATB group (0 (0, 3.5) month vs. 3 (0, 13) months, *p* = 0.028) (Table 1). The case and control groups showed no difference in immunosuppressant or biologics treatment (Table 1) and comorbidities (diabetes mellitus: 4.76% vs. 7.25%, *p* = 1.00; myelodysplastic syndrome: 4.76% vs. 7.25%, *p* = 1.00; and others: 52.38% vs. 52.17%, *p* = 1.00).

#### 3.3.2. Symptoms and Signs

The systemic involvement of BD patients with and without ATB revealed no difference (Table 2), but more ATB patients had TB toxic symptoms like fever (85.7% vs. 49.3%, *p* = 0.003), cough (38.1% vs. 7.2%, *p* = 0.002), expectoration (33.3% vs. 4.3%, *p* = 0.001), and night sweating (38.1% vs. 5.8%, *p* = 0.001).

#### 3.3.3. Laboratory Tests

ATB patients had increased level of erythrocyte sedimentation rate (ESR, 31 (22, 57) vs. 16 (6, 39) mm/h, *p* = 0.004), hypersensitive C reactive protein (hsCRP, 28.32 (8.50, 63.83) vs 10.37 (1.61, 43.59) mg/L, *p* = 0.038), immunoglobulin G (IgG, 12.55 (9.98, 15.61) vs. 9.6 (7.84, 13.13) g/L, *p* = 0.006), complement 3 (C3, 1.3048 ± 0.23713 vs. 1.1664 ± 0.24768 g/L, *p* = 0.036), and complement 4 (C4, 0.2644 ± 0.09852 vs. 0.2213 ± 0.03997 g/L, *p* = 0.039), higher positive rate of T-SPOT.TB (17 (80.95%) vs. 19 (27.54%), *p* = 0.000), and elevated SFC in T-SPOT.TB test (336 (92, 1084) SFC/10^6^PBMC vs. 0 (0, 27) SFC/10^6^PBMC, *p* = 0.000) (Table 2). Complete blood cell count and the liver and kidney function tests revealed no significant difference.

#### 3.3.4. Risk Factors for ATB in BD Patients

The result of logistic regression analysis is shown in Table 3. ESR > 60 mm/h (OR = 13.710, 95% CI (1.101, 170.702), *p* = 0.042), increased IgG (OR = 1.226, 95% CI (1.001, 1.502), *p* = 0.049), and positive T-SPOT.TB (OR = 7.793, 95% CI (1.312, 48.464), *p* = 0.024, for 24-200 SFC/10^6^PBMC; OR = 17.705 (2.503, 125.260), *p* = 0.004, for >200 SFC/10^6^PBMC) were found to be statistically significant.

## 4. Discussion

This study is the first well-designed case-control study investigating the clinical features of BD patients with ATB and exploring the potential risk factors of ATB in BD patients. We demonstrated that BD patients tend to develop pulmonary ATB and have multiple sites involved. Besides presenting with the symptoms related to the infection sites, ATB patients also had systemic symptoms, including fever, night sweating, and unexplained weight loss. Furthermore, logistic regression analysis indicates that ESR > 60 mm/h, increased IgG, and positive T-SPOT.TB are potential risk factors of ATB in BD patients.

Even with thorough examinations, most ATB patients in our study were clinically diagnosed and lacked the microbiological evidence, which is attributed to the high prevalence of TB in China and the complicated nature of the cases referred to our hospital. Microbiological examinations, including bacterial culture, acid-fast staining, and molecular tests, are currently the gold standard for ATB diagnosis. However, the sensitivity of these tests is not satisfactory [22]. In countries with heavy tuberculosis burden, culture- or smear-negative cases are not rare, sometimes even accounting for more than 70% of all ATB cases [20, 23, 24]. Other tests like acute inflammatory markers are not specific, while T-SPOT.TB cannot differentiate ATB from LTBI/PTB [25]. Thus, all patients who were highly suspected of ATB but had negative results in microbiological examinations would receive diagnostic anti-TB treatment for 3 months, and their responses to the treatment were documented to help the clinical diagnosis of ATB. Only by being confirmed by two infectious disease experts would the clinical diagnosis of ATB be accepted. In this way, we believe the clinical diagnosis of ATB in our study is reliable. Besides, our study enrolled BD patients with LTBI, PTB, and non-TB as the control group. Compared with previous case reports of patients mostly culture confirmed [26, 27] and the case-control studies lacking LTBI/PTB patients in the control group [17], our study population is closer to the actual clinical situation.

Generally, patients with ATB are more likely to experience the symptoms related to the site of infection. Therefore, TB infection should be suspected when a BD patient presented with the manifestations rarely occurring in BD, such as productive cough, bone pain, restricted range of motion, and lymphadenopathy. Severe systemic symptoms (fever, night sweating, and unexplained weight loss) are also important clues of ATB. It is challenging to identify active TB in BD patients when they presented with manifestations shared by BD and ATB, such as erythema nodosa, pericarditis, and gastrointestinal ulcers predominant at the ileocecal junction. Laboratory tests can be helpful in this situation. Patients with markedly elevated ESR, hsCRP, IgG, and/or positive T-SPOT.TB should be considered to have ATB, which is consistent with previous case reports [28] and studies [17, 29]. The involvement of lungs in BD generally presents as pulmonary artery thrombosis or aneurysm [30, 31], which could be easily differentiated from tuberculosis by chest CT, while the pulmonary parenchymal involvement in BD is rare. Empiric anti-TB treatment can be considered for patients with the abovementioned clues of ATB. Meanwhile, the response to anti-TB therapy could help the clinical diagnosis or exclusion of ATB.

ESR > 60 mm/h, increased IgG, and positive T-SPOT.TB were found to be potential risk factors for ATB in patients with BD by logistic regression analysis. ESR was reported to be markedly [27] or slightly [32] increased in ATB patients, and increased IgG in ATB patients had also been reported in Wang's case report [28] and Liu's case-control study [17]. However, prospective cohort studies are needed to understand whether the increase of these nonspecific inflammatory markers is the cause or the result of ATB. T-SPOT.TB is an interferon-gamma release assay (IGRA) based on the MTB-specific T cell response [25]. The logistic analysis revealed a higher OR value as spot forming cells (SFC) of T-SPOT.TB increases. Even though T-SPOT.TB could not differentiate LTBI and ATB [25], many studies had shown more robust T cell immune response in ATB patients [20, 33], supporting that positive or markedly increased T-SPOT.TB can be a potential risk factor of ATB in patients with BD.

Notably, our study revealed a smaller dosage and shorter glucocorticoid treatment duration in ATB patients, which is different from previous studies [34]. This may be due to the retrospective design of our study, when patients not excluded for ATB may have avoided using glucocorticoids during the previous treatment. TNF-*α* inhibitor use, though identified as a risk factor of ATB in previous studies [9], revealed no significant difference between cases and controls in our study. On the one hand, this might be related to the limited sample number in this study; on the other hand, this could be attributed to the thorough screening and proper prophylaxis of patients using TNF-*α* inhibitors, according to the guidelines [11, 13, 35, 36] published in the 2000s.

We acknowledge some limitations in our study. First, our sample size was relatively small, and most ATB cases were diagnosed according to clinical criteria rather than culture confirmed, which might introduce a risk of bias. Second, our center is a national referral center for complicated and critical cases, which might induce a potential selection bias, and the extension of the conclusion must be drawn carefully. Third, the risk factors identified in this case-control study are still required to be confirmed by prospective cohort studies in the future. Finally, most (81.0%) of the ATB cases in this study were clinically diagnosed without microbiological evidence. Even though the clinical diagnosis was carefully reviewed and seemed to be plausible in a country with high prevalence of tuberculosis, the diagnosis without a gold standard might introduce a high risk of bias.

## 5. Conclusion

The diagnosis of ATB should be considered when BD patients presented with systemic symptoms like fever, night sweating, and unexplained weight loss, as well as rare presentations of BD such as productive cough, lymphadenopathy, and bone pain. Significantly elevated ESR, hsCRP, IgG, and positive T-SPOT.TB supports the diagnosis of ATB. Furthermore, the markedly increased SFC in T-SPOT.TB indicates a high risk factor of ATB in BD patients.

## Figures and Tables

**Table 1:** Past medical history and medication of BD patients with and without ATB.

	BD with ATB (*n* = 21)	BD without ATB (*n* = 69)	*p*
Sex (male, %)	13 (61.90%)	35 (50.72%)	0.369
Age (*M* ± SD)	36.19 ± 12.46	38.58 ± 12.68	0.450
Past medical history			
Previous contact with ATB patients (%)	3 (14.29%)	2 (2.90%)	0.081
Previous prophylactic treatment of TB (%)	1 (4.76%)	3 (4.35%)	1.000
Evidence of PTB ^a^ (%)	13 (61.90%)	17 (24.64%)	0.002
Previous treatment of BD			
Glucocorticoid			
Maximal dosage (mg/d, median, IQR)^b^	0 (0, 50)	25 (0, 60)	0.093
Duration (months, median, IQR)	0 (0, 3.5)	3 (0, 13)	0.028
Biologics			
Infliximab (%)	2 (9.52%)	2 (2.90%)	0.231
Other TNF-*α* inhibitors (%)	2 (9.52%)	3 (4.35%)	0.587
Immunosuppressant			
CTX (%)	5 (23.81%)	11 (15.94%)	0.515
CsA (%)	0 (0%)	10 (14.49%)	0.109
MTX (%)	1 (4.76%)	3 (4.35%)	1.000
FK506 (%)	1 (4.76%)	2 (2.90%)	0.554
AZA (%)	2 (9.52%)	5 (7.25%)	0.663
Current treatment of BD^c^			
Glucocorticoid dosage (mg/d, median, IQR)^b^	0 (0, 15)	5 (0, 32.5)	0.177
Biologics			
Infliximab (%)	0 (0%)	2 (2.90%)	1.000
Other TNF-*α* inhibitor (%)	1 (4.76%)	0 (0%)	0.233
Immunosuppressant			
CTX (%)	1 (4.76%)	11 (15.94%)	0.281
CsA (%)	1 (4.76%)	7 (10.14%)	0.675
MTX (%)	0 (0%)	0 (0%)	—
FK506 (%)	1 (4.76%)	1 (1.45%)	0.414
AZA (%)	1 (4.76%)	1 (1.45%)	0.414

^a^Evidence of PTB includes past history of TB infection and radiological features indicating PTB in chest CT. ^b^All forms of glucocorticoid were converted to the equivalent dosage of prednisone. ^c^Current use of immunosuppressant and biologics indicates continuous use of the drug for 3 months before hospitalization, and the dosage of glucocorticoid records the maximal dose of glucocorticoid within 2 weeks before hospitalization. TNF-*α*: tumor necrosis factor-*α*; CTX: cyclophosphamide; CsA: ciclosporin A; MTX: methotrexate; FK506: tacrolimus; AZA: azathioprine; LEF: leflunomide; MMF: mycophenolate mofetilhs.

**Table 2:** Clinical presentation and laboratory results of BD patients with and without ATB.

	BD with ATB (*n* = 21)	BD without ATB (*n* = 69)	*p*
Systemic involvement of BD			
Oral ulceration (%)	19 (90.5%)	68 (98.6%)	0.135
Genital ulceration (%)	18 (85.7%)	49 (71.0%)	0.176
Erythema nodosa (%)	12 (57.1%)	30 (43.5%)	0.272
Ocular lesions (%)	4 (19.0%)	25 (36.2%)	0.140
Vascular manifestations (%)	4 (19.0%)	25 (36.2%)	0.140
Gastrointestinal involvement (%)	6 (28.6%)	24 (34.8%)	0.597
CNS involvement (%)	3 (14.3%)	15 (21.7%)	0.548
Symptoms related to TB infection			
Fever (%)	18 (85.7%)	34 (49.3%)	0.003
Cough (%)	8 (38.1%)	5 (7.2%)	0.002
Expectoration (%)	7 (33.3%)	3 (4.3%)	0.001
Night sweating (%)	8 (38.1%)	4 (5.8%)	0.001
Weight loss (%)	13 (61.9%)	28 (40.6%)	0.086
Laboratory tests			
ESR (mm/h, median, IQR)	31 (22, 57)	16 (6, 39)	0.004
hsCRP (mg/L, median, IQR)	28.32 (8.50, 63.83)	10.37 (1.61, 43.59)	0.038
IgG (g/L, median, IQR)	12.55 (9.98,15.61)	9.6 (7.84,13.13)	0.006
IgA (g/L, median, IQR)	2.78 (1.75, 3.66)	2.28 (1.59, 3.04)	0.286
IgM (g/L, median, IQR)	0.91 (0.71, 1.74)	0.93 (0.71, 1.33)	0.575
Positive T-SPOT.TB (%)	17 (80.95%) (*n* = 19)	19 (27.54%) (*n* = 66)	0.000
T-SPOT.TB value (SFC/10^6^PBMC, median, IQR)	336 (92, 1084)	0 (0, 27)	0.000

ESR: erythrocyte sedimentation rate; hsCRP: hypersensitive C reactive protein; IgG: immunoglobulin G; IgA: immunoglobulin A; IgM: immunoglobulin M; SFC: spot-forming cells; PBMC: peripheral blood mononuclear cells.

**Table 3:** Potential risk factors for ATB in BD patients.

	*b*	SE(*b*)	Wald	*p*	OR (95% CI)
ESR (mm/h)					
0-20			4.658	0.097	
20-60	1.719	0.965	3.178	0.075	5.581 (0.843, 36.960)
>60	2.618	1.287	4.141	0.042	13.710 (1.101, 170.702)
IgG (g/L)	0.204	0.104	3.876	0.049	1.226 (1.001, 1.502)
T-SPOT.TB (SFC/10^6^PBMC)					
<24			9.266	0.010	
24-200	2.076	0.921	5.084	0.024	7.793 (1.312, 48.464)
>200	2.874	0.998	8.288	0.004	17.705 (2.503, 125.260)

OR: odds ratio; CI: confidential interval.

## Data Availability

The clinical data used to support the findings of this study are included in the article.

## Supplementary Materials

Supplementary materials The supplementary materials include two tables. Table S1: The diagnostic criteria for different TB infection statuses. Table S2: the detailed information of 21 BD patients diagnosed with ATB.
