# Supplementary material for: Clinical Features and Risk Factors of Active Tuberculosis in Patients with Behçet's Disease
Source: J Immunol Res. 2020 Nov 24;2020:2528676. doi: 10.1155/2020/2528676 (PMC7707958; doi:10.1155/2020/2528676)
Supplement: Supplementary materials — The supplementary materials include two tables. Table S1: The diagnostic criteria for different TB infection statuses. Table S2: the detailed information of 21 BD patients diagnosed with ATB. [file 2528676.f1.pdf]

Supplement materials

Table S1. Diagnosis and categorization criteria for TB

Diagnosis	Criteria
Active tuberculosis (ATB)	1) Symptoms related to TB: cough, expectoration, fever, night sweating, weight loss, etc.<br>2) With or without laboratory results or radiological features supporting TB diagnosis.<br>3) Respond to anti-TB treatment.<br>4) Pathogenic evidence of *Mycobacterium tuberculosis* infection: fast-acid staining, microbiological culture, X-pert, etc.<br>1+4 or 1+2+3 indicates ATB diagnosis, those with 4 were considered microbiologically confirmed ATB while those without were considered clinically diagnosed.
Latent tuberculosis infection (LTBI)	1) No clinical or radiological features of ATB.<br>2) No history or radiological features of previous TB.<br>3) Positive T-SPOT.TB results.<br>1+2+3 indicates LTBI diagnosis.
Previous tuberculosis (PTB)	1) No clinical or radiological features of ATB<br>2) Previous history of TB.<br>3) Radiological features suggestive of previous TB.<br>1 + 2 or 3 indicates PTB diagnosis.

Table S2. The diagnosis of ATB

Case	Sex/Age	Microbiologically confirmed	The diagnosis was made according to	Infection site
1	F/24	Yes	Sputum culture	Lung
2	M/48	Yes	Sputum acid fast staining	Lung
3	M/30	Yes	Bone marrow culture	Lung, ribs, bone marrow
4	M/23	Yes	Sputum culture	Lung
5	M/56	No	History of untreated ATB, symptoms (fever, EN), response to anti-TB treatment	Lymph node
6	M/33	No	Symptoms (fever, EN, cough, expectoration), radiological findings (chest CT revealed bilateral multiple pulmonary tubercles and mediastinal lymphadenopathy), response to anti-TB treatment	Lung
7	M/34	No	Symptoms (fever, night sweating, pharyngalgia, oral and lingual ulcers, epiglottedema), response to anti-TB treatment	Larynx
8	F/56	No	Symptoms (abdominal pain, diarrhea or constipation, cough, expectoration, night sweating), endoscopic findings (colonoscopy revealed multiple longitudinal and oval ulcers in the descending colon and upper rectum), response to anti-TB treatment	Lung, gastrointestinal tract

9	F/38	No	Symptoms (fever, night sweating, chest pain), radiological findings (chest CT revealed a left-sided mass lesion and pleural effusion), response to anti-TB treatment	Lung, peritoneum
10	F/48	No	Symptoms (fever, night sweating, EN, back pain), radiological findings (MRI revealed abnormal signals in the body of vertebrae L5 to S1, with destructed structure of intervertebral discs and stenosis of the intervertebral space), response to anti-TB treatment	Bone and joint
11	M/13	No	Symptoms (fever, abdominal pain), endoscopic findings (colonoscopy showed multiple erosional ulcers with deformation and stenosis of the ileocecal valve), response to anti-TB treatment	Gastrointestinal tract
12	M/23	No	Symptoms (fever), radiological findings (chest CT revealed bilateral pulmonary miliary tubercles and patches), response to anti-TB treatment	Lung
13	F/32	No	Symptoms (oral and genital ulcers, uveitis, neurological symptoms), radiological findings (chest CT revealed a tubercle with cavity and surrounding patches in the right upper lobe), response to anti-TB treatment	Lung
14	F/48	No	History of untreated ATB, symptoms (fever, left cervical lymphadenopathy with fistula formation), response to anti-TB treatment	Lung
15	M/29	No	Symptoms (fever, fatigue, cough, expectoration), radiological findings (chest CT revealed patches in the right upper lobe), response to anti-TB treatment	Lung

16	M/34	No	Symptoms (fever, fatigue, night sweating, weight loss, EN), radiological findings (chest CT revealed bilateral multiple patches and cords; echocardiogram showed pericardial coarctation and cellulosic pericardial exudation), response to anti-TB treatment	Lung, pericardium
17	F/15	No	Symptoms (fever, EN, cervical lymphadenopathy), radiological findings (chest CT showed multiple tubercle and cords in the right lower lobe, and multiple calcified hilar and mediastinal lymphadenopathy), response to anti-TB treatment	Lung
18	M/40	No	Symptoms (cough), radiological findings (chest CT revealed bilateral multiple ground glass opacities and cords-like shadows), response to anti-TB treatment	Lung
19	M/44	No	Symptoms (fever, EN), radiological findings (chest CT revealed bilateral military tubercles), response to anti-TB treatment	Lung
20	M/43	No	History of untreated ATB, symptoms (oral and genital ulcers, folliculitis-like rashes), respond to anti-TB treatment	Unidentified
21	F/49	No	Symptoms (fever, fatigue, night sweating, weight loss, EN), radiological findings (chest CT revealed bilateral multiple patches, ground glass opacities, consolidation and cords-like shadows with partial calcification), response to anti-TB treatment	Lung

EN: erythema nodosum
